# Novel *PRRT2* mutation in an African-American family with paroxysmal kinesigenic dyskinesia

**DOI:** 10.1186/1471-2377-12-93

**Published:** 2012-09-18

**Authors:** Peter Hedera, Jianfeng Xiao, Andreas Puschmann, Dragana Momčilović, Steve W Wu, Mark S LeDoux

**Affiliations:** 1 Department of Neurology, Vanderbilt University, Nashville, TN, USA; 2 Departments of Neurology, and Anatomy and Neurobiology, University of Tennessee Health Science Center, 855 Monroe Avenue, Suite 415 Link Building, Memphis, TN, 38163, USA; 3 Department of Neurology, Skåne University Hospital and Department of Neurology, Lund University, Lund, Sweden; 4 Clinic for Child Neurology and Psychiatry, Medical Faculty University of Belgrade, Belgrade, Serbia; 5 Department of Pediatrics, University of Cincinnati, Cincinnati, OH, USA

**Keywords:** PKD, PRRT2, African-American, ICCA, Hotspot mutation

## Abstract

**Background:**

Recently, heterozygous mutations in *PRRT2* (Chr 16p11.2) have been identified in Han Chinese, Japanese and Caucasians with paroxysmal kinesigenic dyskinesia. In previous work, a paroxysmal kinesigenic dyskinesia locus was mapped to Chr 16p11.2 - q11.2 in a multiplex African-American family.

**Methods:**

Sanger sequencing was used to analyze all four *PRRT2* exons for sequence variants in 13 probands (9 Caucasian, 1 Caucasian-Thai, 1 Vietnamese and 2 African-American) with some form of paroxysmal dyskinesia.

**Results:**

One patient of mixed Caucasian-Thai background and one African-American family harbored the previously described hotspot mutation in *PRRT2* (c.649dupC, p.R217Pfs*8). Another African-American family was found to have a novel mutation (c.776dupG, p.E260*). Both of these variants are likely to cause loss-of-function via nonsense-mediated decay of mutant *PRRT2* transcripts. All affected individuals had classic paroxysmal kinesigenic dyskinesia phenotypes.

**Conclusions:**

Heterozygous *PRRT2* gene mutations also cause paroxysmal kinesigenic dyskinesia in African-Americans. The c.649dupC hotspot mutation in *PRRT2* is common across racial groups.

## Background

Paroxysmal kinesigenic dyskinesia (PKD, OMIM 128000), also known as episodic kinesigenic dyskinesia (EKD1) and paroxysmal kinesigenic choreoathetosis (PKC), is a rare autosomal dominant neurological disorder characterized by recurrent, brief attacks of involuntary movement usually triggered by sudden voluntary movement [1,2]. These attacks usually begin in childhood or early adulthood and may include various combinations of dystonia, chorea, and athetosis affecting the face, trunk, arms and legs. Oftentimes, PKD improves with age and most patients show a favorable response to anticonvulsant medications, particularly carbamazepine or phenytoin [1,2]. Recently, mutations in *PRRT2* (Chr 16p11.2) have been causally associated with both familial and sporadic cases of PKD, infantile convulsions and choreoathetosis (ICCA), benign familial infantile epilepsy (BFIE), paroxysmal exercise-induced dyskinesia (PED), and paroxysmal non-kinesigenic dyskinesia-like (PNKD-like) syndromes in Han Chinese, Japanese and Caucasians [3-11].

PKD is clinically and genetically heterogeneous, and, in at least one British pedigree, does not map to Chr 16 [12]. Work to date suggests that fewer than 50% of patients with primary PKD harbor mutations in *PRRT2 *[6,8]. To expand the genotypic spectrum of *PRRT2* mutations and examine the role of *PRRT2* in other racial groups, we report the clinical and genetic data for 13 probands with paroxysmal dyskinesias including 1 Vietnamese, 1 mixed Caucasian-Thai and 2 African-Americans.

## Methods

All human studies were performed in accordance with institutional review board guidelines at each participating institution, the Helsinki Declaration, and written informed consent for genetic studies and publication of clinical data was obtained from all subjects or, where participants were children, their parents. All genetic and phenotypic analyses and publication of the results were approved by the University of Tennessee Health Science Center Institutional Review Board (#01-07346-XP). Subjects were acquired from outpatient clinics at participating institutions. Clinical diagnoses were made by means of history and examination by one or more board-certified neurologists at each institution. Clinical and genetic details for 13 probands are presented Table 1.

**Table 1:** Clinical details and genetic results for subjects with paroxysmal dyskinesias

**Subject (Diagnosis)**	***PRRT2* Mutation**	**Age/Gender**	**Race**	**Age at onset(y)**	**Family history**	**Attack frequency**	**Attack duration**	**Triggers**	**Involuntary movements**	**Anatomical distribution**	**Response to anticonvulsants**
Family A, II-1 (PKD)	NA	NA/M	African-American	10y	Yes	< 100/day	10-20 sec	SM	D, C	A, L	carbamazepine(+), phenytoin (+)
Family A, III-1 (PKD)	NA	28y/F	African-American	12y	Yes	20-30/day	10-40 sec	SM	D, C	F, A, L	phenytoin (+)
Family A, III-2 (PKD)	NA	25y/F	African-American	10y	Yes	50-75/day	10-15 sec	SM	D, C	A, L	carbamazepine (+)
Family A, III-3 (PKD)	c.776dupG	22y/F	African-American	10y	Yes	30-40/day	10-15 sec	SM	D, C	F, A, L	carbamazepine(+), phenytoin(+)
Family A, III-4 (PKD)	c.776dupG	18y/F	African-American	13y	Yes	20-30/day	10-60 sec	SM	D, C	F, A, L	carbamazepine(+)
Family B (PKD)	c.649dupC	30y/M	African-American	12y	Yes	50/day	10-60 sec	SM	D, C	F, A	phenytoin (+)
Case 7 (PKD)	c.649dupC	27y/M	Caucasian-Thai	21y	*Yes	3-6/day	< 10 sec	SM, S	D	F, A, L	carbamazepine (+)
Case 8 (PED)	None	29y/F	Caucasian	< 28y	No	< 1/day	2-4 hrs	Intense exercise	D	F, A, L	clonazepam (±)
Case 9 (PKD)	None	18y/M	Caucasian	14y	No	5-8/day	< 15 sec	SM	D	F, A, L	carbamazepine (+)
Case 10 (ICCA)	None	19mo/M	Caucasian	7 m	No	>100/day	40-50 sec	SM	D, C, A	F, A, L	carbamazepine (+)
Case 11 (PKD)	None	41y/F	Caucasian	< 33y	Yes	20-25/mo	2-30 min	SM, S	D	F, A, L	piracetam (±) clonazepam (±)
Case 12 (PKD)	None	20y/M	Caucasian	3y	No	6-7/day	5-60 sec	SM, S	D, C, A	F, A, L	carbamazepine (+)
Case 13 (PKD)	None	18y/M	Caucasian	15y	Yes	2-5/day	5-6 sec	SM	D	F, A, L	carbamazepine (+)
Case 14 (PKD)	None	18y/F	Caucasian	15y	No	3-4/day	< 10 sec	SM, S	D, C, A	F, A, L	carbamazepine (+)
Case 15 (PKD)	None	14y/M	Vietnamese	12y	No	10/day	15-60 sec	S	D	A, L	acetazolamide (+)
Case 16 (PKD)	None	26y/F	Caucasian	16y	No	30/day	20-30 sec	SM	D	A	phenytoin (+)
Case 17 (PNKD)	None	6y/F	Caucasian	6 m	Yes	2-10/mo	3-60 min	Fatigue, sleep deprivation	D	A, L	NA

Involuntary movements: D-dystonia, C-chorea, and A-athetosis. Anatomical distribution: F-face, A-arm, and L-leg.

Triggers: SM-sudden movement and S-stress. NA, DNA or clinical detail not available. Response to anticonvulsants:

+, good to excellent response and ±, partial response, *, early childhood seizures on maternal (Thai) side of the family.

DNA was extracted from peripheral blood leucocytes using Roche’s DNA Isolation Kit for Mammalian Blood (Indianapolis, IN, USA). DNA quantity and quality were analyzed with a NanoDrop ND-1000 spectrophotometer (Wilmington, DE, USA) and agarose gel electrophoresis. With Primer3 (frodo.wi.mit.edu), four pairs of PCR primers were designed to encompass the four *PRRT2* exons and flanking intronic regions (Additional file 1 Table S1). For Sanger sequencing, PCR was performed using 50 ng of template DNA, 1X PCR buffer, 2.5 mM MgCl_2_ and 200 nM of each primer in a 20-μl reaction volume. The following cycling conditions were employed: 95°C for 15 min; 35 cycles at 95°C for 15 s, 60°C for 15 s, and 72°C for 45 s; and 72°C for 10 min. After agarose gel confirmation, 5 μl of the PCR products were cleaned using ExoSAP-IT® (United States Biochemical, Cleveland, OH, USA). Then, 1-2 μl of the purified PCR products were sequenced in the forward and reverse directions on the Applied Biosystems 3130XL Genetic Analyzer (Carlsbad, CA, USA). Control DNA samples (100 African-American and 100 Caucasian) were sequenced for detection of newly-identified *PRRT2* mutations.

## Results

**Figure 1:**
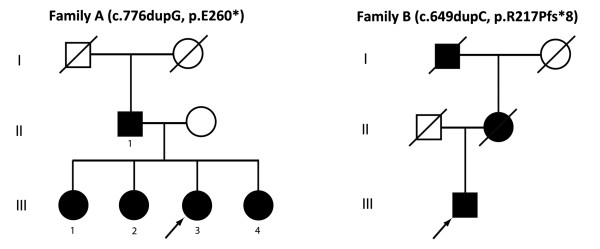
**African-American PKD pedigrees.** Males are represented by squares, females by circles. Affected individuals are represented by filled/black symbols and asymptomatic family members by empty/white symbols. Symbols of deceased individuals are slashed. Probands are denoted with arrows.

Among 13 index cases with paroxysmal dyskinesias, two different mutations in three families were identified. A novel mutation was found in African-American Family A (Figure 1, c.776dupG, p.E260*). This mutation was not found in 100 African-American or 100 Caucasian normal controls. The proband was a 22-year-old female (Figure 1, III-3), who noticed the first attack of choreiform and dystonic movements in her hands and arms at age 12. Subsequent episodes also included dystonia in her legs and face. Her father and all three sisters have similar clinical features during attacks with dystonia in the face, arms and legs, along with chorea in the hands. Although DNA specimens were not available from her father and two older sisters, the c.776dupG mutation was confirmed in her youngest sister (III-4). All affected family members responded to either carbamazepine or phenytoin. Two of the three family members currently taking phenytoin did not tolerate carbamazepine due to sedative effects.

The previously reported hotspot mutation (c.649dupC, p.R217Pfs*8) was found in African-American Family B (Figure 1) and an individual of mixed Caucasian-Thai background. The c.649dupC variant was not found in 100 African-American or 100 Caucasian normal controls. Case 7 had late-onset (>20 y) but otherwise classic carbamazepine-responsive PKD. Prior to initiation of therapy with carbamazepine, sudden movements were more likely to precipitate dystonic posturing when the patient was under psychological stress. Attacks often consisted of dystonic posturing of the left arm in abduction along with cervical dystonia. Occasionally, similar attacks affected the right side of the body. Although his Thai mother had no history of PKD, ICCA or BFIE and was found to be neurologically normal, Sanger sequencing revealed that she was a carrier, and several of her family members reportedly had infantile seizures.

No sequence variants were identified in the remaining 10 probands (9 Caucasian, 1 Vietnamese) with PED, ICCA, PKD or PNKD, 3 of whom had a positive family history. All but two of these individuals had early-onset (< 20 y) paroxysmal dyskinesias. Age of onset, attack frequency and attack duration were much more variable among the mutation-negative cases in comparison to the patients with *PRRT2* mutations.

## Discussion

Candidate regions for PKD and ICCA were mapped to Chr 16 over a decade ago. PKD was linked to a 15.8 cM region flanked by markers D16S685 and D16S503 on Chr 16q13-q22.1 with a maximum LOD score of 3.66 at D16S419 in a large Indian family [13]. This candidate region was telomeric to a locus identified in Japanese families with PKD [14], but showed overlap with a region identified in an African-American family with PKD [15]. A candidate region for ICCA had also been mapped to the pericentromeric region of Chr 16 in French [16] and Chinese [17] families.

Just recently, several distinct loss-of-function frameshift mutations leading to protein truncation or nonsense-mediated decay in proline-rich transmembrane protein 2 (PRRT2) have been associated with PKD in numerous Han Chinese families [3-6]. A much smaller percentage of cases were associated with missense mutations (e.g., c.796C > T, p.R266W; c.913 G > A, p.G305R) [4,6]. In addition to classic carbamazepine-responsive PKD, the phenotypic spectrum of *PRRT2* mutations includes cases of ICCA, BFIE, some “PNKD-like” syndromes, and PED [6-11]. *PRRT2* is located on Chr 16p11.2, within the ICCA, Japanese PKD, and African-American candidate regions but outside the Indian PKD candidate region. The association of *PRRT2* genotypes with specific neurological phenotypes may become apparent with the publication of additional well-characterized cases.

PRRT2 is a cell surface protein containing two predicted transmembrane domains and highly expressed in the developing nervous system, particularly the cerebellum [3]. Our study has shown that novel and hotspot mutations in *PRRT2* are associated with classic PKD in African-Americans. The c.776dupG and c.649dupC mutations are heterozygous SNindels (single nucleotide insertions or deletions) predicted to cause nonsense-mediated decay of mutant transcripts rather than expression of a truncated protein [18,19]. SNindels occur at an estimated frequency of 0.887 per 10 kb of genomic DNA with more than half occurring in regions with mononucleotide repeats [19]. The novel c.776dupG mutation is located within a 6 nucleotide (nt) poly-G tract and the c.649dupC hot spot mutation is in a 9 nt poly-C tract. SNindels within regions of mononucleotide repeats may arise from replication slippage [19].

## Conclusions

The novel c.776dupG mutation and c.649dupC hot spot mutation identified in our African-American families with classic PKD expands the molecular and racial spectrums of *PRRT2* mutations. As evidenced from our patient of mixed Caucasian-Thai descent, the penetrance of *PRRT2* mutations may depend on the origin of the normal or wild-type allele. Finally, a significant percentage of patients with PKD and ICCA do not harbor mutations in coding regions of *PRRT2*.

## Abbreviations

PKD, Paroxysmal kinesigenic dyskinesia; EKD1, Episodic kinesigenic dyskinesia; PKC, Paroxysmal kinesigenic choreoathetosis; ICCA, Infantile convulsions and choreoathetosis; BFIE, Benign familial infantile epilepsy; PED, Paroxysmal exercise-induced dyskinesia; PNKD, Paroxysmal non-kinesigenic dyskinesia.

## Competing interests

The authors declare that they have no competing interests.

## Authors’ contributions

MSL designed the study, examined research subjects, contributed to the initial draft of the manuscript, and analyzed genetic data. PH extracted DNA from blood specimens, examined research subjects and contributed to the initial draft of the manuscript. JX performed Sanger sequencing, analyzed genetic data, and contributed to the initial draft of the manuscript. AP, DM, and SW examined subjects. All authors reviewed and critiqued the manuscript.

## Pre-publication history

The pre-publication history for this paper can be accessed here:

http://www.biomedcentral.com/1471-2377/12/93/prepub

## Supplementary Material

Additional file 1 **Table S1.***PRRT2* Sequencing Primers. Click here for file
