# Supplementary material for: Novel PRRT2 mutation in an African-American family with paroxysmal kinesigenic dyskinesia
Source: BMC Neurol. 2012 Sep 18;12:93. doi: 10.1186/1471-2377-12-93 (PMC3460747; doi:10.1186/1471-2377-12-93)
Supplement: Additional file 1 — Table S1.PRRT2 Sequencing Primers. [file 1471-2377-12-93-S1.doc]

**sUPPORTING INFORMATION**

**Supplementary Table 1.** *PRRT2* Sequencing Primers

| Primer name | **Sequence (5’→3’)** | Locus | Usage | Product (bp) |
| --- | --- | --- | --- | --- |
| PRRT2_E1F | ctccagacacccgcattc | NC_000016.9 29823735-718 | Exon 1 sequencing | 418 (with PRRT2_1F) |
| PRRT2_E1R | ccggggacttaagaaggag | NC_000016.9 29823318-336 |  |  |
| PRRT2_E2F1 | ggatccatgcagagaggaga | NC_000016.9 29825371-352 | Exon 2 sequencing | 690 (with PRRT2_2F1) |
| PRRT2_E2R1 | gacccatgccaagaaacagt | NC_000016.9 29824682-701 |  |  |
| PRRT2_E2F2 | tagggagctctggttgaagg | NC_000016.9 29824859-840 | Exon 2 sequencing | 689 (with PRRT2_2F2) |
| PRRT2_E2R2 | gcaaaggaaaccccaacttt | NC_000016.9 29824171-190 |  |  |
| PRRT2_E4F | ggaggtttccagaagtgcag | NC_000016.9 29826243-224 | Exons 3 & 4 sequencing | 714 (with PRRT2_4F) |
| PRRT2_E3R | ctcccttacccgccatctat | NC_000016.9 29825530-549 |  |  |
